# First Isolation and Molecular Characterization of Umatilla Virus (Sedoreoviridae, Orbivirus) in Brazil

**DOI:** 10.3390/v16071050

**Published:** 2024-06-28

**Authors:** Landeson Barros, Sandro Silva, Ana Cecília Cruz, Eliana da Silva, Ana Lúcia Wanzeller, Valéria Carvalho, Jannifer Chiang, Lívia Martins

**Affiliations:** Department of Arbovirology and Hemorrhagic Fevers, Evandro Chagas Institute, Ananindeua 67030-000, Pará, Brazil; landesonbarros@iec.gov.br (L.B.); sandrosilva@iec.gov.br (S.S.); anacecilia@iec.gov.br (A.C.C.); elianasilva@iec.gov.br (E.d.S.); anawanzeller@iec.gov.br (A.L.W.); valeriacarvalho@iec.gov.br (V.C.); liviamartins@iec.gov.br (L.M.)

**Keywords:** *Orbivirus*, Umattila virus, *Turdus fumigatus*, genomic description

## Abstract

In this study, we provide a genomic description of the first isolation of the Umattila virus (UMAV) in Brazil. The virus was obtained from the blood of a bird (*Turdus fumigatus*) and isolated in a C6/36 cell culture. The viral genome contains ten segments, and its organization is characteristic of viruses of the genus *Orbivirus* (family *Sedoreoviridae*). The coding region of each segment was sequenced, demonstrating the nucleotide identity with UMAV. The phylogenetic inference results were in line with these findings and demonstrated the formation of two distinct monophyletic clades containing strains isolated around the world, where our isolate, belonging to the same clade as the prototype strain, was allocated to a different subclade, highlighting the genetic divergence between them. This work reports the first isolation of UMAV in Brazil, and due to the scarcity of information on this viral agent in the scientific literature, it is essential to carry out further studies to better understand its epidemiology, dispersion, and, in particular, its interactions with vertebrate hosts, vectors, and the environment.

## 1. Introduction

The *Orbivirus* genus is one of the six genera of the family *Sedoreoviridae*, which has the highest number of species, including viruses of the serogroup Changuinola [1]. The viral particle has an icosahedral morphology, no viral envelope, and a genome composed of 10 double-stranded RNA (dsRNA) segments, which encode seven structural proteins and three non-structural proteins [2]. Serological tests, such as neutralization testing and complement fixation, have been used to identify different orbivirus species, and molecular methods now carry out a more accurate classification [3].

Several orbiviruses are classified as arboviruses and are isolated from different arthropods (culicoides, sandflies, and ticks) and vertebrate hosts [4]. Several species have great economic significance due to their severe impact on the livestock industry, such as the African horse sickness virus (AHSV) and the Bluetongue virus (BTV) [5]. Other orbiviruses are epidemiologically relevant, causing epizootic diseases such as *Peruvian horse sickness virus* (PHSV), *Equine encephalosis virus* (EEV), *Palyam virus* (PALV), *epizootic hemorrhagic disease virus* (EHDV), and *Tribe virus* (TRBV), which are associated with infections in humans [2,6,7].

The continuous advances in molecular studies, in particular nucleotide sequencing, have allowed for a better understanding of the relationship between orbiviruses, especially those belonging to the species *Umatilla virus*, which is composed of four serotypes: *Umatilla virus*, (UMAV), *Minnal virus* (MINV), *Netivot virus* (NETV), *Llano Seco virus* (LLSV), and the new member *Stretch Lagoon Virus* (SLOV) [7]. The first isolation of UMAV occurred in the city of Umatilla, Oregon, USA, obtained from a pool of *Culex pipiens* collected on 30 July 1969, and later from a sample collected from a bird of the species *Passer domesticus* in the State of Texas in 1967 [8]. The present study aims to report the first viral isolation of UMAV in Brazil, more precisely in the Amazon region, and its molecular characterization.

## 2. Materials and Methods

### 2.1. Viral Isolation in Cell Culture

The UMAV isolate (AV13752/AN813360) was obtained from the blood of a bird (*Turdus fumigatus*) without signs of disease, captured in the National Forest of Carajás in Parauapebas, Pará State, Brazil, in 2015. The National Forest of Carajás is an environmental conservation area of approximately 411,948.87 hectares, in which the most representative plant typologies are the Open Ombrophylous Forest, the dense Ombrophylous Forest, and the Ferruginous Rupestre Field [9]. In addition, it has a rich fauna characterized by a diversity of vertebrate species [10]. This area has a mineral extraction project and is constantly undergoing environmental changes. Our group has been conducting studies on arboviruses in this area, assessing the possible impacts on the transmission cycles of these agents. Wild animals such as rodents, marsupials, and birds have been captured for arbovirus research, as have hematophagous arthropods (mosquitoes).

For viral isolation, we used 2 cells lines commonly used for the isolation of arboviruses: C6/36 cell culture (*Aedes albopictus*—ATCC: CRL 1660) and VERO (kidney of *Cercopithecus aethiops*—ATCC: CCL 81). Briefly, 25 µL of the undiluted blood was added to the C6/36 and VERO cell cultures, which were then maintained in Leibowit’z L-15 and 199 media, respectively; supplemented with L-glutamine; and supplemented with 2% Fetal Bovine Serum (FBS), 2.95% tryptose phosphate (HiMedia laboratories LLC, Kelton, PA, USA) and antibiotics (penicillin 100 UL/mL and streptomycin 100 UL/mL). We monitored the cell monolayers daily for ten days to identify a cytopathic effect (CPE), and if positive, the material was submitted to metagenomic analysis to identify and characterize the viral isolate [11,12].

### 2.2. RNA Extraction, Nucleotide Sequencing, and Phylogenetic Analysis

To extract RNA, 140 µL of the infected cell culture was processed using the QIAamp Viral RNA Mini Kit. The first- and second-strand cDNA was synthesized using the SuperScript^TM^ VILO^TM^ MasterMix and the NEBNext^®^ Second Strand Synthesis Module, respectively. The reaction was then purified using the PureLink^®^ purification kit (Thermo Fisher Scientific Inc., Waltham, MA, USA).

We used the Nextera XT DNA kit (Illumina Inc., San Diego, CA, USA) to prepare the genomic library from cDNA, and nucleotide sequencing was carried out using the methodology described in the Preparation Kit on the Miniseq platform (Illumina Inc., San Diego, CA, USA), using the MiniSeq High Output kit—300 Cycles (Illumina Inc., San Diego, CA, USA), and the paired-end method at the department of Arbovirology and Hemorrhagic Fevers of the Evandro Chagas Institute, Ministry of Health, Brazil. 

The reads were assembled using the De Novo Assembler methodology with the SPAdes [13] and IDBA-UD [14] programs to recover the viral genome. The Diamond program [15] was used to compare all contigs with the RefSeq virus protein database available at NCBI, and the results were visualized in the Megan 6 program [16]. The inspection and annotation of putative ORF genes was performed using Geneious v.9.1.6 software (Biomatters, New Zealand). All ten segments were analyzed by InterProScan 5 to detect protein domains using the UniProtKB protein sequence database [17].

Multiple Sequence Alignment (MSA) was performed using the Mafft v.7 program [18]. Before the phylogenetic analysis, ProtTest was applied to select the best amino acid substitution model [19]. The maximum likelihood (ML) method [20] was used to reconstruct the phylogenetic tree, implemented in RaxML v.8.2.4 [21]. In addition, bootstrap analysis [22] was performed on 1000 replicates to determine the reliability of the tree topology. Phylogeny visualization was performed using the FigTree v.1.4.4 software (http://tree.bio.ed.ac.uk/software/figtree/ accessed on 06/02/2024). The data set did not have a root sequence, so the methodology of midpoint rooting, a tool available in the phylogeny visualization program, was used. After assessing and editing the phylogeny, a file with the extension “SVG” (Scalable Vector Graphics) was generated to edit and manipulate the image using the Inkscape v.1.1 program (https://inkscape.org/ accessed on 16/11/2021).

### 2.3. Reassortment Analysis

Bootscan analysis, using SimPlot v3.5.1 software, was performed to identify possible reassortment between segments of UMAV. Bootscan is a computational method used in molecular phylogenetics to detect potential recombination or reassortment events in viral genomes. It works by analyzing the similarity between a query sequence, in this case the AV13752/AN813360 (NCBI ID OQ749748 to OQ749757), and a set of reference sequences along the genome. By comparing the query sequence with multiple reference sequences in sliding windows, Bootscan calculates the phylogenetic support (bootstrap values) for each window position. High bootstrap values suggest that the query sequence is more closely related to one reference sequence, indicating potential recombination or reassortment breakpoints.

## 3. Results

### 3.1. Virus Isolation in Cell Culture and Molecular Analysis

During the daily monitoring of the cell culture, we identified a discreet ECP in the C6/36 cells on the 7th day after the inoculation of the bird blood, characterized by some points of cell aggregation, probably caused by the UMAV replication. During the 10 days of VERO cell monitoring, we did not observe any cellular changes in the cell monolayer (ECP).

Proceeding with the identification and molecular analysis of the virus isolation obtained in the C6/36 cells, the generation of contigs in the assembly step, and our comparison with public databases, we identified the presence of 10 RNA segments (NCBI ID OQ749748 to OQ749757) with nucleotide and amino acid identity with UMAV values ranging from 61.5% to 86.6% and 60.0% to 98.9%, respectively (NCBI ID HQ842619 to HQ842628). Lower identity values were also observed compared to other members of the *Orbivirus* genus (Table 1). The UMAV from Brazil, showed identity with others UMAV (Supplementary File S1).

The size of the segment genomes ranged from 3920 to 843 nt among the obtained sequences, and it was possible to recover the complete protein coding region (ORFs) for segments 1, 4, 5, 6, 7, 8, 10 and the partial protein coding region for segments 2, 3, and 9. Through analysis of the functional protein domains, we identified four structural proteins (VP2, VP3, VP5, and VP7) and six non-structural proteins (VP1, VP4, VP6, NS1, NS2, and NS3) (Table 2). In segments 2 (OQ749749), 3 (OQ749750), and 9 (OQ749756), 8, 40, and 51 nt are missing in ORF 3′, respectively.

**Figure 1:**
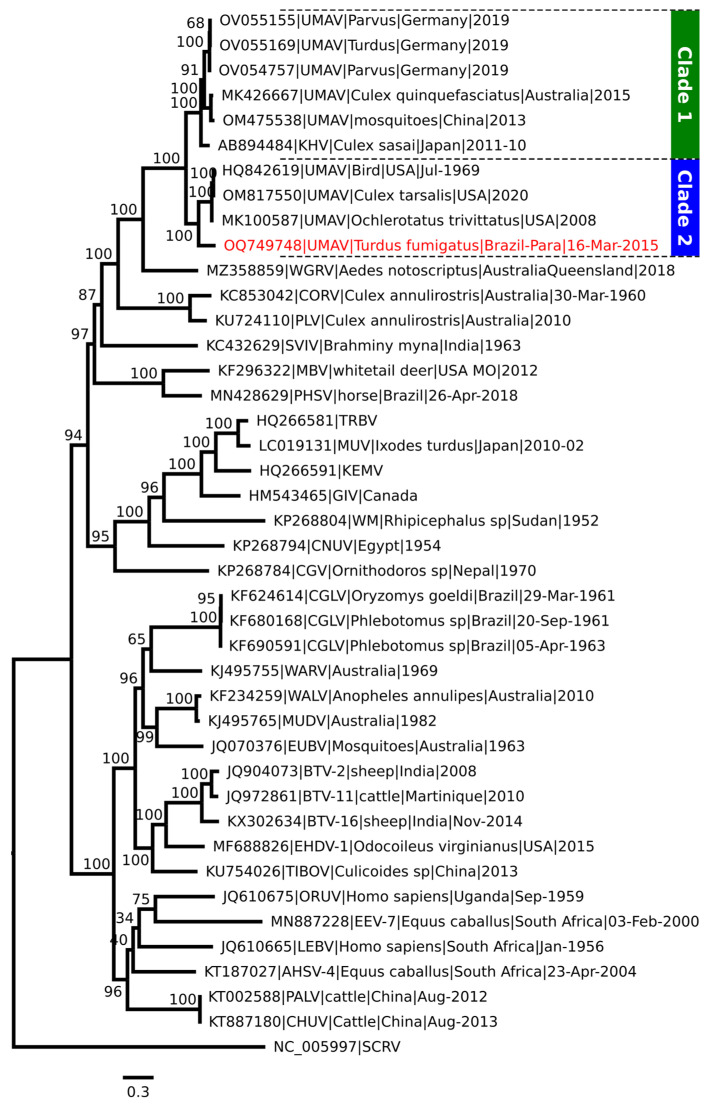
Phylogenetic inference of different species of the *Orbivirus* genus. Phylogenetic tree generated from Maximum Likelihood (ML) methodology based on the amino acid sequences of the segment encoding the polymerase (VP1), constructed using the LG + I + G4 substitution model, which best represents this set of data. The sample identified in this study is highlighted in red. The numbers at each major node of the tree correspond to the bootstrap values in percentage (1000 replicates). The scale bar corresponds to nucleotide divergence per site between the sequences.

For phylogenetic inference, we used the amino acid sequence of the VP1 protein, which encodes the polymerase, because it is the most conserved between the Orbiviruses, identifying a monophyletic clade formed by different UMAV strains. However, two distinct monophyletic clades were formed by strains originating from countries in Europe and Asia (Old World) and North and South America (New World), with the CORV and PLV being more external to these clades (Figure 1).

### 3.2. Reassortment Analysis

**Figure 2:**
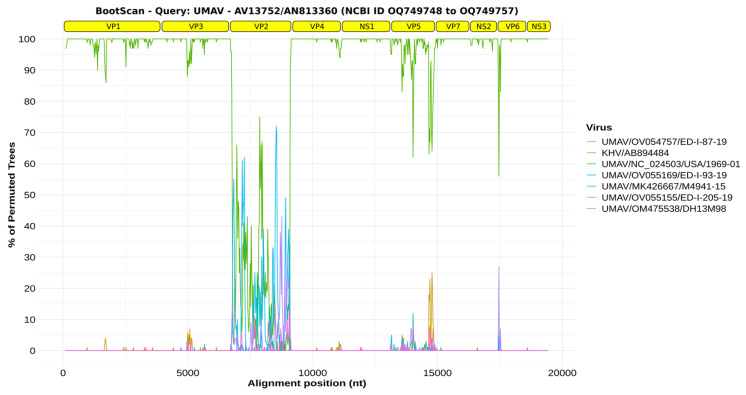
Reassortment analysis results obtained using Bootscan. The figure illustrates the Bootscan analysis of the query ten-segment sequence of Umatilla virus strain AV13752/AN813360 (NCBI ID OQ749758). The identification of all segments is presented at the top of the chart. The analysis employed the following parameters: Window: 200 bp, Step: 20 bp, GapStrip: On, Reps: 100, Kimura (2-parameter), T/t: 2.0, Neighbor-Joining. The values of the permutation tests are expressed as percentages, and the alignment positions are indicated in nucleotides (nt).

The genome of the ten segments of the UMAV clade identified in the phylogenetic tree was used to verify reassortment events. This analysis did not include two UMAV isolates because they needed to have all ten segments (UMAV-2020 was missing VP3 and NS3 [NCBI ID OM817550 to OM817557], and UMAV-IA08 was missing VP2, VP7, and NS3 [NCBI ID MK100587 to MK100593]). Each segment was aligned individually and concatenated. The sequence of AV13752/AN813360 was used as a reference sequence in the Bootscan analysis. The Bootscan plots below demonstrate high permuted trees between UMAV isolate USA1969/01 and the reference sequence with almost all segments, except for segment three, which encodes the VP2 protein (Figure 2).

## 4. Discussion

This genomic description of the UMAV strain first isolated in Brazil (AV13752/AN813360) is compatible with those already described in the literature for the genus *Orbivirus* (*Sedoreoviridae* family) [1,2,3,23], a condition that contributed to its inclusion within this group of arboviruses with significant impacts on public health, especially animal health [5,24].

Phylogenetic studies conducted to classify viruses of the *Sedoreoviridae* family, both at the genus and species level, have used RNA-dependent viral polymerase (VP1) due to it being highly conserved among orbiviruses [4,6,25]. This information guided the use of the VP1 protein during our analysis, as the correlation matrix built from it was able to ratify the existing genetic relationship between the UMAV isolate AV13752/AN813360 and the other orbiviruses, mainly with the UMAV strains.

This close relationship with UMAV was evidenced in the phylogenetic tree. However, it led to the formation of two distinct monophyletic clades and the grouping of the isolated AV13752/AN813360 with the strains originating from the American continent (New World). Regarding the clades, a probable explanation may be associated with the existing genetic variations in the VP2 protein, as demonstrated in the Bootscan analysis. VP2 and VP2 homologous proteins can be encoded by segments 2, 3, 4, and 5 and are designated VP2, VP3 (YUOV, SCRV), VP4 (BRDV segment 4), and GIV segment 5 [6].

In all UMAV sequences available in NCBI, the VP2 is present in segment 3, the third-largest segment of the 10, with a length of 2411 nt (NC_024505). Most studies do not focus on VP2 in UMAV; however, in BTV, the VP2 protein corresponds to the outer capsid, which constitutes the primary target of the host immune response and neutralization antigen region [26], and it is one of the most variable segments [27].

The UMAV identified in this study showed high variability in VP2 compared to other UMAVs, with deficient amino acid identity (60%) and evidence of permutation according to our Bootscan analysis. In this case, two possibilities can be considered: this virus accumulated several mutations overtime to escape the immune response, or it underwent a reassortment event with another UMAV that has not been identified yet.

Another interesting factor is the question of how UMAV arrived in Brazil, since there is a geographic limitation between countries that have already detected the UMAV. In practice, what is observed are few reports involving the biological, ecological, and epidemiological characteristics of UMAV; however, for other arboviruses, the transmission cycle is something already well documented in the literature, especially the involvement of birds because they are considered one of the primary amplifiers of these viral agents [28,29], and consequently, they play a vital role in the spread of arboviruses. 

When moving in a viremic period, these animals serve as a food source for uninfected arthropod vectors in their new destinations. For this reason, birds are often considered responsible for introducing arboviruses to new geographic areas [30,31]. Molecular data have shown that the genetic similarity between viruses associated with birds in different geographic locations is connected through migratory routes, thus highlighting the importance of birds in arbovirus transport [32,33,34]. In addition, other factors reinforce this information: (i) the vectors are relatively resident and, in theory, do not have the potential to travel long distances, unlike migratory birds [35,36]; (ii) the eggs of vectors that transmit arboviruses hatch less frequently in colder regions [37,38]; (iii) birds can travel great distances very quickly [39,40]. 

Information synthesized by Brown and Valerie [31] demonstrates that many arbovirus isolations have occurred more frequently in resident birds than migratory ones and that the virus they transport occurs around their nesting territories. The fact is that the isolation of UMAV in the region of Canaã dos Carajás not only confirms this information, since the isolate was obtained from a resident bird of the species *Turdus fumigatus*, but also allows us to infer that the coexistence between species of migratory birds and those residing in the same ecosystem can influence the transmission cycle of this arbovirus, since the State of Pará has routes used by migratory birds for resting, feeding, and reproduction [41]. 

It should also be considered that the success of birds in transporting arboviruses depends on a primordial factor: the existence of competent vectors for transmission after they arrive in new locations [42,43]. The involvement of mosquitoes of the *Culex* genus in the transmission of UMAV is known [8], as is the involvement of mosquitoes of the *Aedes* genus in the transmission of other orbiviruses [6,44], and their presence has already been found in studies conducted in the Canaã dos Carajás region [45]. This condition makes them potential vectors for maintaining the UMAV transmission cycle in the Canaã dos Carajás region.

## 5. Conclusions

Based on all the evidence identified in this study, it was possible to confirm the presence of UMAV in the forest area of Canãa dos Carajás, Pará state, Brazil. However, a more extensive surveillance effort is needed in this region to determine if this virus is established in the area or if this was an isolated case. This would involve studying other birds and arthropods in the region to determine the possible presence and spread of UMAV in the region. However, the most acceptable hypothesis is that UMAV could have come to Brazil through a migratory bird.

## Figures and Tables

**Table 1:** Nucleotide (nt) and amino acid (aa) sequence identity between isolated *Umatilla Virus* (AV13752/AN813360) and other orbiviruses.

Virus	VP1	VP2	VP3	VP4	VP5	VP6	VP7	NS1	NS2	NS3
	nt/aa	nt/aa	nt/aa	nt/aa	nt/aa	nt/aa	nt/aa	nt/aa	nt/aa	nt/aa
BTV	51.6/47.5	26.6/9.8	47.0/35.8	50.1/45.0	44.3/32.3	34.5/21.5	38.3/24.4	33.0/19.4	30.5/16.3	29.9/14.8
CORV	56.8/58.0	29.1/15.0	52.8/47.8	53.2/50.1	52.2/46.9	39.0/23.2	45.2/41.5	41.9/30.8	40.6/30.7	34.2/20.0
KHV	74.9/89.0	60.6/58.0	79.5/95.1	71.0/77.9	73.4/82.8	73.6/64.6	81.0/91.7	76.0/87.1	70.6/77.1	67.0/66.5
MOBV	55.8/51.4	29.9/11.8	51.0/43.5	50.0/42.1	48.8/37.8	38.9/25.7	46.4/32.3	41.5/26.7	37.3/23.3	28.3/19.4
PLV	51.2/47.0	26.0/7.5	47.0/35.6	50.4/43.9	43.2/29.9	32.0/18.1	40.3/25.3	34.5/20.3	32.3/17.8	27.9/11.8
SVIV	55.8/53.9	28.5/12.3	51.2/448	48.7/44.4	47.9/34.6	33.9/24.8	48.4/36.1	35.8/22.0	38.5/27.7	27.7/18.2
UMAV	80.6/94.7	61.5/60.0	83.8/97.7	77.0/85.0	76.2/89.4	82.8/76.7	86.6/98.9	80.9/93.6	81.4/94.6	83.1/92.0
YUOV	54.8/50.3	29.1/11.7	49.5/40.7	48.0/42.8	48.2/35.7	34.5/23.4	45.4/30.4	38.9/22.8	35.6/24.1	29.4/17.0

Legend: Bluetongue virus (BTV—JQ904064 to JQ904073); Corriparta virus (CORV—KC853042 to KC853051); Koyama Hill virus (KHV—AB894484 to AB894493); Mobuck virus (MOBV—KF296322 to KF296331); *Palyam virus* (PALV—KT002588 to KT002597); Sathuvachari virus (SVIV—KC432629 to KC432638); Umatilla virus (UMAV—HQ842619 to HQ842628); and Yunnan Orbivirus (YUOV—AY701509 to AY701518).

**Table 2:** Genomic description of 10 segments of UMAV (AV13752/AN813360) as compared with the prototype from NCBI (HQ842619 to HQ842628).

Segment *	Protein	Mapping Reads	Coverage	Genome Size (nt)	5′ UTR Length	ORF Size (nt)	Situation ORF	Protein (aa)	Weight (KDa)	3′ UTR Length	NCBI ID
1	RNA Pol Orbiviruses (VP1)	577	21	3920	11	3900	Complete	1299	147,807	9	OQ749748
2	Inner layer core VP3 Orbiviruses (T2)	451	23	2728	18	2710	Incomplete	902	103,146	NA	OQ749749
3	Capsid VP2 Orbiviruses (VP2)	827	50	2311	17	2294	Incomplete	764	89,838	NA	OQ749750
4	Orbiviruses VP4 (Cap)	1899	134	1979	4	1947	Complete	648	75,829	28	OQ749751
5	Orbiviruses NS1 (TuP)	621	47	1771	13	1743	Complete	580	66,995	15	OQ749752
6	Capsid VP5 Orbiviruses	486	43	1641	27	1590	Complete	529	5884	24	OQ749753
7	BTV NS2	486	53	1318	60	1224	Complete	407	46,073	34	OQ749754
8	Orbiviruses VP7 capsid (T13)	237	31	1074	8	1056	Complete	351	38,953	10	OQ749755
9	Orbiviruses VP6 (Hel)	394	50	999	24	975	Incomplete	324	34,632	NA	OQ749756
10	Orbiviruses NS3	93	15	843	12	795	Complete	264	29,362	36	OQ749757

* The segment order was based on the size and not the encoding protein(s). T = 2 icosahedral capsid protein (T2), capping 2-OMTase viral (Cap), hydrophobic tubular protein (TuP); T = 13 icosahedral capsid protein (T13) and orbivirus helicase (Hel). NA: Not applicable.

## Data Availability

Dataset available upon request to authors.

## Supplementary Materials

The following supporting information can be downloaded at: https://www.mdpi.com/article/10.3390/v16071050/s1, Supplementary File S1: Alignment of different strains of Umatilla virus

## References

[1] Matthijnssens J., Attoui H., Bányai K., Brussaard C.P.D., Danthi P., del Vas M., Dermody T.S., Duncan R., Fāng Q., Johne R. (2022). ICTV Virus Taxonomy Profile: *Sedoreoviridae* 2022. J. Gen. Virol..

[2] Dilcher M., Hasib L., Lechner M., Wieseke N., Middendorf M., Marz M., Koch A., Spiegel M., Dobler G., Hufert F.T. (2012). Genetic Characterization of Tribeč Virus and Kemerovo Virus, Two Tick-Transmitted Human-Pathogenic Orbiviruses. Virology.

[3] Zerbini F.M., Siddell S.G., Lefkowitz E.J., Mushegian A.R., Adriaenssens E.M., Alfenas-Zerbini P., Dempsey D.M., Dutilh B.E., García M.L., Hendrickson R.C. (2023). Changes to Virus Taxonomy and the ICTV Statutes Ratified by the International Committee on Taxonomy of Viruses (2023). Arch. Virol..

[4] Attoui H., Jaafar F.M., Belhouchet M., Aldrovandi N., Tao S., Chen B., Liang G., Tesh R.B., de Micco P., de Lamballerie X. (2005). *Yunnan orbivirus*, a New Orbivirus Species Isolated from *Culex tritaeniorhynchus* Mosquitoes in China. J. Gen. Virol..

[5] Wilson A.J., Mellor P.S. (2009). Bluetongue in Europe: Past, Present and Future. Philos. Trans. R. Soc. B Biol. Sci..

[6] Belaganahalli M.N., Maan S., Maan N.S., Tesh R., Attoui H., Mertens P.P.C. (2011). Umatilla Virus Genome Sequencing and Phylogenetic Analysis: Identification of Stretch Lagoon Orbivirus as a New Member of the Umatilla Virus Species. PLoS ONE.

[7] MacLachlan N.J., Guthrie A.J. (2010). Re-Emergence of Bluetongue, African Horse Sickness, and Other Orbivirus Diseases. Vet. Res..

[8] Karabatsos N. (1985). International Catalogue of Arboviruses, Including Certain Other Viruses of Vertebrates.

[9] ICMBio (2014). Relatório Anual de Rotas e Áreas de Concentração de Aves Migratórias No Brasil.

[10] Martins F.D., Castilho A.F., Campos J., Hatano F.M., Rolim S.G. (2012). Fauna da Floresta Nacional de Carajás.

[11] Barbosa M.L., Rocco I.M., Felippe J.M.M.S., Cruz A.S. (1993). Growth and Maintenance of *Aedes albopictus* Cell Line, Clone C6/36, in Diferrent Media. Rev. Inst. Adolfo Lutz.

[12] Igarashi B. (1978). Isolation of a Singh’s Aedes Albopictus Cell Clone Sensitive to Dengue and Chikungunya Viruses. J. Gen. Virol..

[13] Bankevich A., Nurk S., Antipov D., Gurevich A.A., Dvorkin M., Kulikov A.S., Lesin V.M., Nikolenko S.I., Pham S., Prjibelski A.D. (2012). SPAdes: A New Genome Assembly Algorithm and Its Applications to Single-Cell Sequencing. J. Comput. Biol..

[14] Peng Y., Leung H.C.M., Yiu S.M., Chin F.Y.L. (2012). IDBA-UD: A de Novo Assembler for Single-Cell and Metagenomic Sequencing Data with Highly Uneven Depth. Bioinformatics.

[15] Buchfink B., Xie C., Huson D.H. (2014). Fast and Sensitive Protein Alignment Using DIAMOND. Nat. Methods.

[16] Huson D.H., Auch A.F., Qi J., Schuster S.C. (2007). MEGAN Analysis of Metagenomic Data. Genome Res..

[17] Jones P., Binns D., Chang H.-Y., Fraser M., Li W., McAnulla C., McWilliam H., Maslen J., Mitchell A., Nuka G. (2014). InterProScan 5: Genome-Scale Protein Function Classification. Bioinformatics.

[18] Katoh K., Standley D.M. (2013). MAFFT Multiple Sequence Alignment Software Version 7: Improvements in Performance and Usability. Mol. Biol. Evol..

[19] Abascal F., Zardoya R., Posada D. (2005). ProtTest: Selection of Best-Fit Models of Protein Evolution. Bioinformatics.

[20] Myung I.J. (2003). Tutorial on Maximum Likelihood Estimation. J. Math. Psychol..

[21] Stamatakis A. (2014). RAxML Version 8: A Tool for Phylogenetic Analysis and Post-Analysis of Large Phylogenies. Bioinformatics.

[22] Felsenstein J. (1985). Confidence Limits on Phylogenies: An Approach Using the Bootstrap. Evolution.

[23] Silva S.P., Dilcher M., Weber F., Hufert F.T., Weidmann M., Cardoso J.F., Carvalho V.L., Chiang J.O., Martins L.C., Lima C.P.S. (2014). Genetic and Biological Characterization of Selected Changuinola Viruses (*Reoviridae*, *Orbivirus*) from Brazil. J. Gen. Virol..

[24] Mellor P.S., Hamblin C. (2004). African Horse Sickness. Vet. Res..

[25] Belaganahalli M.N., Maan S., Maan N.S., Nomikou K., Pritchard I., Lunt R., Kirkland P.D., Attoui H., Brownlie J., Mertens P.P.C. (2012). Full Genome Sequencing and Genetic Characterization of Eubenangee Viruses Identify Pata Virus as a Distinct Species within the Genus *Orbivirus*. PLoS ONE.

[26] Fukusho A., Ritter G.D., Roy P. (1987). Variation in the Bluetongue Virus Neutralization Protein VP2. J. Gen. Virol..

[27] Maan S., Maan N.S., Samuel A.R., Rao S., Attoui H., Mertens P.P.C. (2007). Analysis and Phylogenetic Comparisons of Full-Length VP2 Genes of the 24 Bluetongue Virus Serotypes. J. Gen. Virol..

[28] Stamm D.D. (1966). Relationships of Birds and Arboviruses. Auk.

[29] Degallier N., da Rosa A.P.A.T., da Silva J.M.C., Rodrigues S.G., da Costa Vasconcelos P.F., da Rosa J.F.S.T., da Silva G.P., da Silva R.P. (1992). Aves Como Hospedeiras de Arbovírus Na Amazônia Brasileira. Bol. do Mus. Para. Emílio Goeldi.

[30] Georgopoulou I., Tsiouris V. (2008). The Potential Role of Migratory Birds in the Transmission of Zoonoses. Vet. Ital..

[31] Brown C.R., O’Brien V.A. (2011). Are Wild Birds Important in the Transport of Arthropod-Borne Viruses?. Ornithol. Monogr..

[32] Weaver S.C., Hagenbaugh A., Bellew L.A., Gousset L., Mallampalli V., Holland J.J., Scott T.W. (1994). Evolution of Alphaviruses in the Eastern Equine Encephalomyelitis Complex. J. Virol..

[33] Young D.S., Kramer L.D., Maffei J.G., Dusek R.J., Backenson P.B., Mores C.N., Bernard K.A., Ebel G.D. (2008). Molecular Epidemiology of Eastern Equine Encephalitis Virus, New York. Emerg. Infect. Dis..

[34] Cilnis M.J., Kang W., Weaver S.C. (1996). Genetic Conservation of Highlands J Viruses. Virology.

[35] Maciel-de-Freitas R., Neto R.B., Gonçalves J.M., Codeço C.T., Lourenço-de-Oliveira R. (2006). Movement of Dengue Vectors between the Human Modified Environment and an Urban Forest in Rio de Janeiro. J. Med. Entomol..

[36] Milby M.M., Reisen W.K., Reeves W.C. (1983). Intercanyon Movement of Marked *Culex tarsalis* (Diptera: Culicidae)1. J. Med. Entomol..

[37] Reisen W.K., Reeves W.C. (1990). Bionomics and Ecology of *Culex tarsalis* and Other Potential Mosquito Vector Species. Epidemiology and Control of Mosquito Borne Arboviruses in California, 1943–1987.

[38] Reisen W.K., Fang Y., Lothrop H.D., Martinez V.M., Wilson J., O’connor P., Carney R., Cahoon-Young B., Shafii M., Brault A.C. (2006). Overwintering of West Nile Virus in Southern California. J. Med. Entomol..

[39] Jourdain E., Gauthier-Clerc M., Bicout D., Sabatier P. (2007). Bird Migration Routes and Risk for Pathogen Dispersion into Western Mediterranean Wetlands. Emerg. Infect. Dis..

[40] Stutchbury B.J.M., Tarof S.A., Done T., Gow E., Kramer P.M., Tautin J., Fox J.W., Afanasyev V. (2009). Tracking Long-Distance Songbird Migration by Using Geolocators. Science.

[41] ICMBio (2019). Relatório de Rotas e Áreas de Concentração de Aves Migratórias No Brasil.

[42] Owen J., Moore F., Panella N., Edwards E., Bru R., Hughes M., Komar N. (2006). Migrating Birds as Dispersal Vehicles for West Nile Virus. Ecohealth.

[43] Agarwal A., Parida M., Dash P.K. (2017). Impact of Transmission Cycles and Vector Competence on Global Expansion and Emergence of Arboviruses. Rev. Med. Virol..

[44] Tangudu C.S., Charles J., Hurt S.L., Dunphy B.M., Smith R.C., Bartholomay L.C., Blitvich B.J. (2019). Skunk River Virus, a Novel Orbivirus Isolated from *Aedes trivittatus* in the United States. J. Gen. Virol..

[45] Oliveira C.F. (2018). Relatório de Gestão do Exercício 2017.

